# Extraction of Information Related to Drug Safety Surveillance From Electronic Health Record Notes: Joint Modeling of Entities and Relations Using Knowledge-Aware Neural Attentive Models

**DOI:** 10.2196/18417

**Published:** 2020-07-10

**Authors:** Bharath Dandala, Venkata Joopudi, Ching-Huei Tsou, Jennifer J Liang, Parthasarathy Suryanarayanan

**Affiliations:** 1 IBM Research Yorktown Heights, NY United States

**Keywords:** electronic health records, adverse drug events, natural language processing, deep learning, information extraction, adverse drug reaction reporting systems, named entity recognition, relation extraction

## Abstract

**Background:**

An adverse drug event (ADE) is commonly defined as “an injury resulting from medical intervention related to a drug.” Providing information related to ADEs and alerting caregivers at the point of care can reduce the risk of prescription and diagnostic errors and improve health outcomes. ADEs captured in structured data in electronic health records (EHRs) as either coded problems or allergies are often incomplete, leading to underreporting. Therefore, it is important to develop capabilities to process unstructured EHR data in the form of clinical notes, which contain a richer documentation of a patient’s ADE. Several natural language processing (NLP) systems have been proposed to automatically extract information related to ADEs. However, the results from these systems showed that significant improvement is still required for the automatic extraction of ADEs from clinical notes.

**Objective:**

This study aims to improve the automatic extraction of ADEs and related information such as drugs, their attributes, and reason for administration from the clinical notes of patients.

**Methods:**

This research was conducted using discharge summaries from the Medical Information Mart for Intensive Care III (MIMIC-III) database obtained through the 2018 National NLP Clinical Challenges (n2c2) annotated with drugs, drug attributes (ie, strength, form, frequency, route, dosage, duration), ADEs, reasons, and relations between drugs and other entities. We developed a deep learning–based system for extracting these drug-centric concepts and relations simultaneously using a joint method enhanced with contextualized embeddings, a position-attention mechanism, and knowledge representations. The joint method generated different sentence representations for each drug, which were then used to extract related concepts and relations simultaneously. Contextualized representations trained on the MIMIC-III database were used to capture context-sensitive meanings of words. The position-attention mechanism amplified the benefits of the joint method by generating sentence representations that capture long-distance relations. Knowledge representations were obtained from graph embeddings created using the US Food and Drug Administration Adverse Event Reporting System database to improve relation extraction, especially when contextual clues were insufficient.

**Results:**

Our system achieved new state-of-the-art results on the n2c2 data set, with significant improvements in recognizing crucial drug−reason (F1=0.650 versus F1=0.579) and drug−ADE (F1=0.490 versus F1=0.476) relations.

**Conclusions:**

This study presents a system for extracting drug-centric concepts and relations that outperformed current state-of-the-art results and shows that contextualized embeddings, position-attention mechanisms, and knowledge graph embeddings effectively improve deep learning–based concepts and relation extraction. This study demonstrates the potential for deep learning–based methods to help extract real-world evidence from unstructured patient data for drug safety surveillance.

## Introduction

### Background

An electronic health record (EHR) is the systematized collection of electronically stored health information of patients and the general population in a digital format [1]. Clinical notes in EHRs summarize interactions that occur between patients and health care providers [2]. These notes include observations, impressions, treatments, drug use, adverse drug events (ADEs), and other activities arising from each interaction between the patient and the health care system. Extracting useful information such as ADEs from these notes and alerting caregivers at the point of care has the potential to improve patient health outcomes.

An ADE is commonly defined as “an injury resulting from medical intervention related to a drug” [3]. ADEs are a major public health concern and one of the leading causes of morbidity and mortality [4]. Studies have shown the substantial economic burden of these undesired effects [5,6]. Although drug safety and efficacy are tested during premarketing randomized clinical trials, these trials may not detect all ADEs because such studies are often small, short, and biased by the exclusion of patients with comorbid diseases. With the limited information available when a drug is marketed, postmarketing surveillance has become increasingly important. Spontaneous reporting systems, such as the US Food and Drug Administration Adverse Event Reporting System (FAERS) [7], are monitoring mechanisms for postmarketing surveillance that enable both physicians and patients to report ADEs. However, previous studies [8-10] have exposed various inadequacies with such systems, including underreporting, reporting biases, and incomplete information, prompting researchers to explore additional sources to detect ADEs from real-world data.

Several efforts have been made to extract ADEs automatically from disparate information sources, including EHRs [11-13], spontaneous reporting systems [14-16], social media [17-20], search queries on the web via search engine logs [21,22], and biology and chemistry knowledge bases [23-25]. Furthermore, the clinical natural language processing (NLP) community has organized several open challenges such as the 2010 Informatics for Integrating Biology & the Bedside/Veterans Affairs NLP Challenge [26], Text Analysis Conference 2017 Adverse Drug Reactions Track [27], and BioCreative V Chemical Disease Relation task [28]. Recently, 2 such challenges, Medication and Adverse Drug Events from Electronic Health Records (MADE 1.0) [29] and the 2018 National NLP Clinical Challenges (n2c2) Shared Task Track 2 [30], were organized to extract *drugs*, drug attributes, *ADEs*, *reasons* for prescribing drugs, and their relations from clinical notes. The results from these 2 challenges showed that deep learning techniques outperform traditional machine learning techniques for this task, and significant improvement is still required for *drug−{ADE, reason}* relation extraction. Specifically, the organizers of these challenges hypothesized that models that can effectively incorporate the larger context to capture long-distance relations or leverage knowledge to capture implicit relations will likely improve the performance of future systems.

Considering these conclusions, we developed a joint deep learning–based relation extraction system that helps in extracting long-distance relations through a position-attention mechanism and implicit relations through external knowledge from the FAERS. To the best of our knowledge, no previous research has been conducted on using the position-attention mechanism and domain-specific knowledge graph embeddings in ADE detection.

### Relevant Literature

#### Adverse Drug Event Detection

From the viewpoint of NLP, effective techniques for entity and relation extraction are fundamental requirements in automatic ADE extraction. Entity and relation extraction from text has traditionally been treated as a pipeline of 2 separate subtasks: named entity recognition (NER) and relation classification. Previous studies employed traditional machine learning techniques [31-34], such as conditional random fields (CRF) [35] for NER and support vector machines [36] for relation classification. Several recent approaches [37-44], developed on MADE 1.0 [29] and 2018 n2c2 Shared Task Track 2 [30] data sets, employed deep learning techniques, such as bidirectional, long short-term memory–conditional random fields (BiLSTM-CRFs) [45], for NER and convolutional neural network (CNN) [46] for relation classification, and showed numerous advantages resulting in better performance and less feature engineering. However, there is an inevitable error propagation issue with pipeline-based methods because of the following:

1. NER relying on sequence-labeling techniques suffers from lossy representation when there are overlapping annotations on entities. For example, in “she was on *furosemide* and became *hypotensive* requiring *norepinephrine*,” *hypotensive* is an *ADE* with respect to *furosemide* but a *reason* with respect to *norepinephrine*.
2. NER approaches usually take an input context window that may not contain the necessary information to determine the appropriate label (ie, *ADE, reason,* no label). For example, in “Patient reports *nausea*. Started on *ondansetron,*” the identification of *nausea* as a *reason* requires information from both sentences.
3. Signs or symptoms are only labeled as *ADE* or *reason* if they are related to a drug (ie, not all signs or symptoms in the clinical note are annotated). This makes the corpus less suitable to train an effective relation classification model as it misses negative candidate pairs for *drug*−{*ADE, reason*} relations.

To address the first 2 issues, we previously proposed a joint method that outperformed the pipeline method for concept and relation extraction on a similar data set (MADE 1.0) [37]. In a separate study, Li et al [47] proposed a joint method using multitask learning [48] and made similar observations. To address the third issue, which was introduced with the n2c2 data set, Wei et al [38] proposed a novel label-encoding scheme to jointly extract *ADE*, *reason*, drug attributes, and their relations.

#### Attention-Based Relation Extraction

The attention mechanism allows neural networks to selectively focus on specific information [49-51]. This has proven to be effective for NLP problems with long-distance dependencies such as NER and relation extraction. Zhou et al [52] proposed an attention-based BiLSTM network and demonstrated its effectiveness in selectively focusing on words that have decisive effects on relation classification. Next, Zhang et al [53] extended the attention mechanism to help networks not only focus on words based on the semantic information of the sentence but also the global positions of entities within the sentence. Recently Dai et al [54] introduced a position-attention mechanism for joint extraction of entities and overlapping relations. The position-attention mechanism builds on self-attention by focusing on both the global dependencies of the input and tokens of the target entities of interest for relation extraction. Recent research [37,55] on ADE extraction showed the benefits of self-attention mechanisms in pipeline-based methods, specifically for relation classification. However, to the best of our knowledge, no previous work has focused on using self-attention or position-attention mechanisms for joint extraction of entities and relations for ADE extraction.

#### Knowledge-Aware Relation Extraction

Several approaches [56-59] in the open domain have shown that incorporating embeddings learned from knowledge bases benefit deep learning–based relation classification. These embeddings are typically learned using translation-based methods such as TransE [60], TransH [61], and TransR [62]; walk-based methods such as DeepWalk [63] and node2vec [64]; or neural network–based methods such as large-scale information network embedding (LINE) [65] and bipartite network embedding [66].

Clinical notes are typically written for medical professionals. Hence, a certain degree of medical knowledge is assumed by the authors, which is not explicitly expressed in the text. This is especially true for relations between clinical findings and drugs, where a drug could either cause (*ADE*) or treat (*reason*) a clinical finding. In our previous study [37], we showed that augmenting knowledge base features such as proportional report ratio and reporting odds ratio calculated from the FAERS into deep learning models can benefit relation classification. Recently, Chen et al [67] proposed a hybrid clinical NLP system by combining a general knowledge-based system using the Unified Medical Language System (UMLS) and BiLSTM-CRF for concept extraction and attention-BiLSTM for relation classification. However, to the best of our knowledge, no previous work has focused on using knowledge graph embeddings generated from the FAERS for joint extraction of entities and relations for ADE extraction.

## Methods

### Data Set

**Figure 1:**
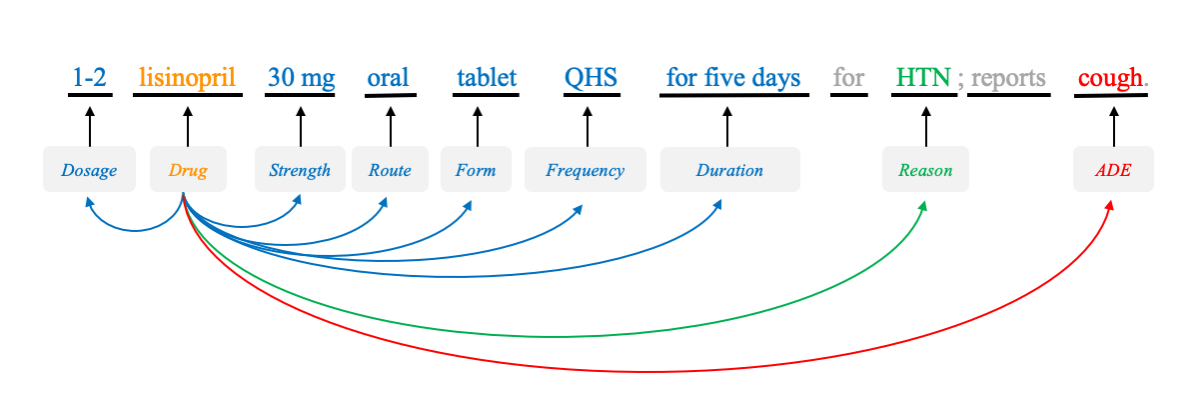
An illustration with annotations for entities and relations. ADE: adverse drug event; HTN: hypertension; QHS: every night at bedtime.

The n2c2 data set consists of 505 deidentified clinical narratives, of which 303 and 202 narratives were released as train and test data sets, respectively. Each narrative was manually annotated with drug-centric entities, including *drugs*, their attributes (*strength*, *form*, *frequency*, *route*, *dosage*, and *duration*), *ADEs*, *reasons*, and relations between drugs and other entities (*drug−*{attributes, *ADE*, *reason*}). *Drug−*{attributes} represent 6 different types of relations: *drug−*{*strength*, *form*, *frequency*, *route*, *dosage*, *duration*}. Figure 1 presents an example with annotations. Tables 1 and 2 present the statistical overview of the annotated entities and relations.

**Table 1:** Entities in the data set.

Entity type	Number of annotations		Example	Description
	Train, n (%)	Test, n (%)		
Drug	16,225 (31.84)	10,575 (32.13)	Coumadin	Name of the drug
Strength	6691 (13.13)	4230 (12.85)	5 mg	Strength of the drug
Form	6651 (13.05)	4359 (13.24)	Tablet	Form of the drug
Frequency	6281 (12.32)	4012 (12.19)	Daily	Frequency of the drug
Route	5476 (10.75)	3513 (10.67)	By mouth	Route in which the drug is administered
Dosage	4221 (8.28)	2681 (8.14)	1	Dosage of the drug
Duration	592 (1.16)	378 (1.15)	For 5 days	Duration of the drug
ADE^a^	959 (1.88)	625 (1.90)	Rash	Adverse reaction of the drug
Reason	3855 (7.57)	2545 (7.73)	Constipation	Indication if it is an affliction that a physician is actively treating with a drug
Total	50,951 (100.00)	32,918 (100.00)	N/A^b^	N/A

^a^ADE: adverse drug event.

^b^Not applicable.

**Table 2:** Relations in the data set.

Relation type	Relations		Intersentential relations		Example^a^
	Train, n (%)	Test, n (%)	Train, n (%)	Test, n (%)	
Drug−strength	6702 (18.44)	4244 (18.09)	80 (1.19)	59 (1.39)	*Lisinopril* 1×*5 mg* tablet orally daily for 7 days
Drug−form	6654 (18.31)	4374 (18.64)	259 (3.89)	144 (3.29)	*Lisinopril* 1×5 mg *tablet* orally daily for 7 days
Drug−frequency	6310 (17.36)	4034 (17.19)	372 (5.90)	238 (5.90)	*Lisinopril* 1×5 mg tablet orally *daily* for 7 days
Drug−route	5538 (15.24)	3546 (15.11)	199 (3.59)	149 (4.20)	*Lisinopril* 1×5 mg tablet *orally* daily for 7 days
Drug−dosage	4225 (11.62)	2695 (11.49)	135 (3.20)	102 (3.78)	*Lisinopril1*×5 mg tablet orally daily for 7 days
Drug−duration	643 (1.80)	426 (1.80)	34 (5.4)	43 (10.0)	*Lisinopril* 1×5 mg tablet orally daily for *7 days*
Drug−ADE^b^	1107 (3.05)	733 (3.10)	254 (22.94)	139 (18.9)	Patient is experiencing *muscle pain*, secondary to *statin* therapy for coronary artery disease
Drug−reason	5169 (14.22)	3410 (14.53)	1638 (31.69)	1088 (31.91)	Patient is experiencing muscle pain, secondary to *statin* therapy for *coronary artery disease*
Total	36,348 (100.00)	23,462 (100.00)	2971 (8.17)	1947 (8.30)	N/A^c^

^a^Italics indicate entities participating in the specified relation type.

^b^ADE: adverse drug event.

^c^Not applicable.

### Preprocessing

Sentence boundary detection (SBD) and tokenization are often treated as solved problems in NLP and carried out using off-the-shelf toolkits such as Apache Natural Language Toolkit [68], Explosion AI spaCy [69] or the Stanford CoreNLP toolkit [70]. However, these are still difficult and critical problems [71] in the clinical domain because (1) sentence ends are frequently indicated by layout and not by punctuation and (2) white space is not always present to indicate token boundaries (eg, *50 mg*). To address these issues, we incorporated domain-specific rules sensitive to low-level features such as capitalization, text-wrap properties, indentation, and punctuation into the spaCy tokenizer and SBD models. These custom rules are provided in Multimedia Appendix 1.

### Representation Learning

#### Static Word Representations

Word embedding is a text vectorization technique that transforms words or subwords into vectors of real numbers. Pretrained word embeddings created using Word2Vec [72], Glove [73], and fastText [74] have been broadly used to initialize deep learning architectures for NLP tasks and have shown substantial improvement over random initialization. Recent research [75] showed that NER performance is significantly affected by the overlap between the pretrained word embedding vocabulary and the vocabulary of the target NER data set. Thus, we used Word2Vec with skip-gram to pretrain word embeddings over the Medical Information Mart for Intensive Care III (MIMIC-III) [76] with the default parameters provided in a study by Mikolov et al [72].

#### Contextualized Word Representations

A well-known limitation of word embedding methods is that they produce a single representation of all possible meanings of a word. To tackle these deficiencies, advanced approaches have attempted to model the word’s context into a vector representation. Embeddings from Language Models (ELMo) [77] is a prominent model that generates contextualized word representations by combining the internal states of different layers in a neural language model. Bidirectional Enconder Representations from Transformers (BERT) [78] furthered this idea by training bidirectional transformers [50] using subwords. Contextualized embeddings are particularly useful for clinical NER as entities (eg, *cold* as low temperature versus infection) have different meanings in different contexts. Recent research [79] showed that deep learning architectures with contextualized embeddings pretrained on a large clinical corpus achieve state-of-the-art performance on several clinical NER data sets. Inspired by these, we trained contextualized representations using ELMo on MIMIC-III. Detailed explanations of ELMo and training parameters are provided in Multimedia Appendix 2.

#### Knowledge Representations

**Figure 2:**
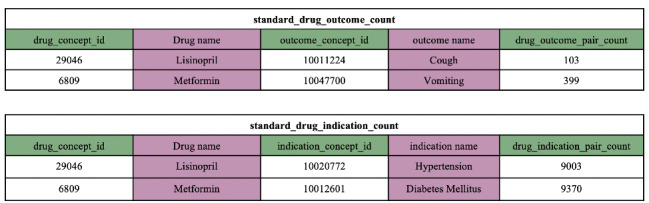
Excerpts from the standard drug outcome count and standard drug indication count tables from adverse event open learning through universal standardization.

To introduce medical knowledge, we built knowledge representations on the FAERS, a database for postmarketing drug safety monitoring. Specifically, we used 2 tables from Adverse Event Open Learning through Universal Standardization (AEOLUS) [14], a curated and standardized FAERS resource, to generate 2 separate graph embeddings. As shown in Figure 2, *standard drug_outcome count* contains case frequencies for drug outcomes, including *ADEs*, and *standard drug indication count* contains case frequencies for drug indications (ie, *reasons)*.

Let *G=(D,O,E)* be a weighted bipartite network, where *D* and *O* denote the set of *drug concept id* and *outcome concept id* in *standard drug outcome count,* and 
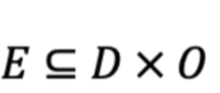
 defines the interset edges. *D_i_* and *O_j_* denote the *i^th^* and *j^th^* vertex in *D* and *O* respectively, where *i*={1,2, … ,|*D*|} and *j*={1,2, … ,|*O*|}. Each edge 
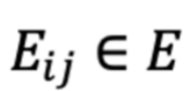
 carries a frequency *f_ij_* provided by the *drug outcome pair count* field in *standard drug outcome count*, indicating the strength between the connected vertices *D_i_* and *O_j_*; if *D_i_* and *O_j_* are not connected, *f_ij_* is set to zero. To integrate this knowledge into our proposed architecture, we computed token-level embeddings by transforming *G* to *G’* as follows:

Given a *drug concept id* (RxNorm) or *outcome concept id* (Medical Dictionary for Regulatory Activities) from AEOLUS, we mapped it to its concept unique identifiers (CUIs) in UMLS [80] and obtained a set of tokens from all CUI variants. Let *d*={*d_1_, d_2_, …., d_L_*} and *o*={*o_1,_ o_2_, …., o_M_*} represent all unique drug and outcome tokens obtained from mapping all 
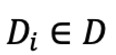
 and 
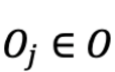
. Let 
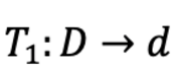
 and 
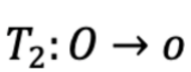
 represent 2 multivalued functions that associate each element in the set of *drug concept id* and *outcome concept id* to a set of tokens. Let *G’=(d,o,e)* be a weighted bipartite graph and each edge 
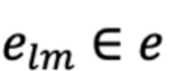
 of *G’* is associated with a nonnegative weight *w_lm_* indicating the strength between the drug token *d_l_* and the outcome token *o_m_*. We calculated *w_lm_* as token-level co-occurrence between *d_l_* and *o_m_* normalized for the drug token *d_l_*:


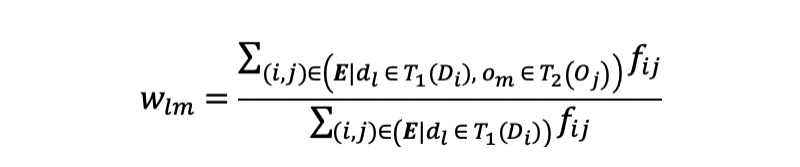


In *w_lm,_* the numerator represents the sum of frequencies of all *drug concept id* and *outcome concept id* pairs that contain drug token *d_l_* and outcome token *o_m_* and the denominator represents the sum of frequencies of all pairs whose *drug concept id* contains the drug token *d_l_*.

From the generated bipartite weighted graph *G’*=(*d,o,e*), we used the LINE approach to generate *drug-adverse* knowledge embeddings. We used LINE because (1) relations between drugs and other concepts in the FAERS form a weighted bipartite graph with a long-tail distribution of vertex degrees and (2) it helps in embedding implicit connectivity relations between vertices of the same type. Similarly, we generated *drug-reason* knowledge embeddings from the *standard drug indication count* table. Detailed explanations of LINE and training parameters are provided in Multimedia Appendix 2.

### Architecture

**Figure 3:**
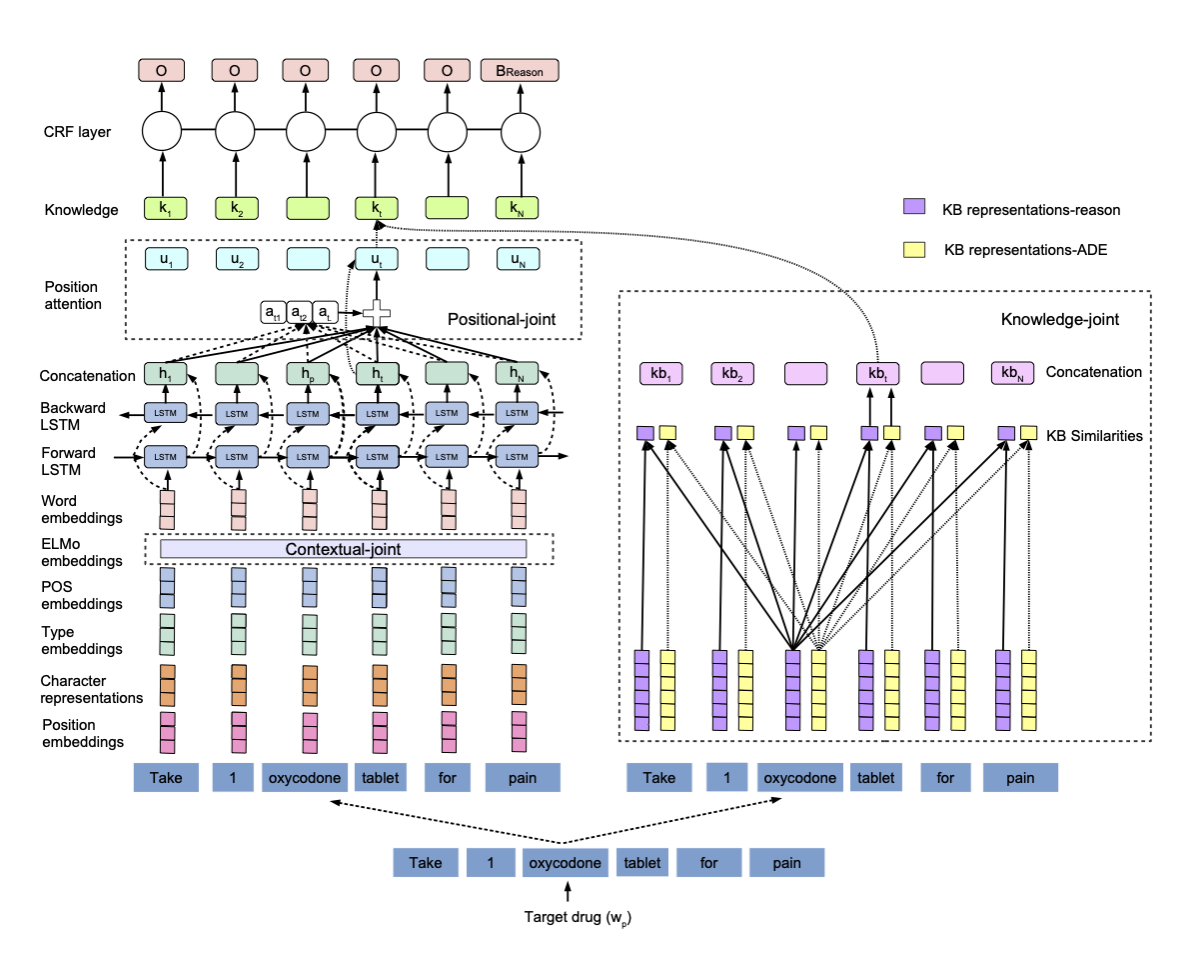
Canonical architecture of the proposed system. ADE: adverse drug event; BReason: beginning of reason annotation; CRF: conditional random field; ELMo: Embeddings from Language Models; KB: knowledge base; LSTM: long short-term memory; POS: part-of-speech.

In the following sections, we present our system, illustrated in Figure 3, in an incremental fashion: *joint method*, *contextual-joint*, *positional-joint*, and *knowledge-joint*. A detailed explanation of the deep learning architecture, BiLSTM-CRF [81], and input embeddings used in this system is included in the Multimedia Appendix 3.

#### Joint Method

We developed a *drug recognition model* followed by 2 joint *drug-centric relation extraction models* (*drug−*{attributes} and *drug−*{*ADE*, *reason*}), as explained in the following sections.

#### Drug Recognition Model

We modeled drug recognition as a sequence-labeling task using BiLSTM-CRF and a beginning, inside, and outside of a drug mention (BIO) tagging scheme. The input layer of the BiLSTM-CRF takes word, character, and part-of-speech embeddings. The word embeddings were obtained using Word2Vec representations generated using MIMIC-III. The character and part-of-speech embeddings were initialized randomly. We used CNNs [46] to encode a character-level representation for a word.

#### Drug-Centric Relation Extraction Models

**Figure 4:**
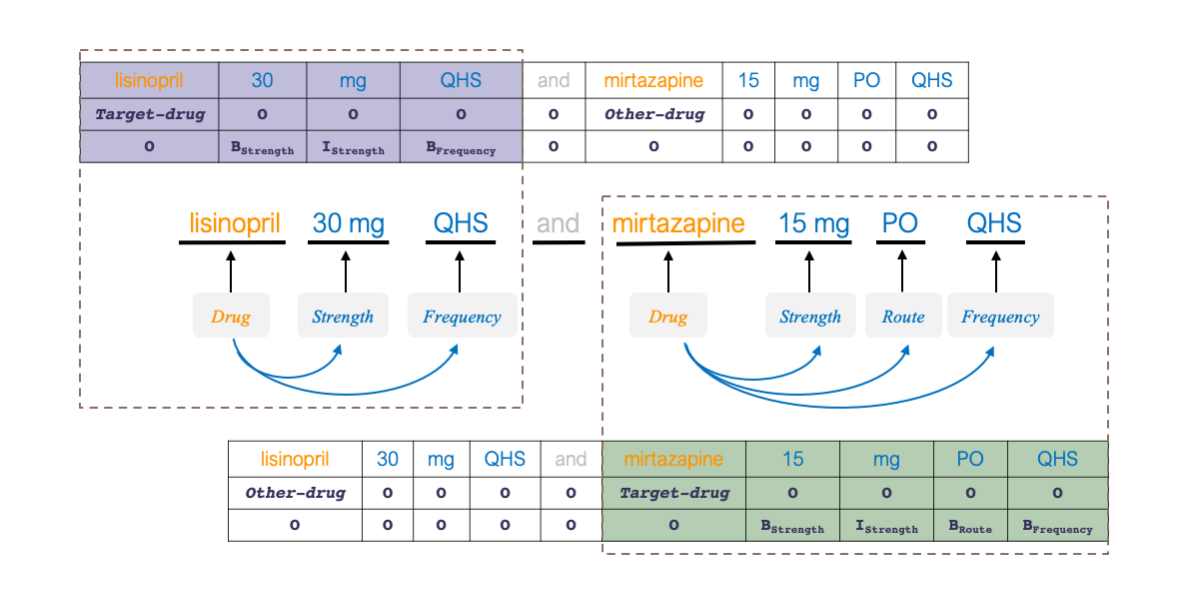
Label-encoding scheme used in drug-centric relation extraction models. B: beginning; I: inside; PO: orally; QHS: every night at bedtime.

To extract entities and relations jointly, we used the encoding scheme proposed in [38], which takes annotated sentences and produces drug-centric sequences for a specified *target-drug*. For sentences containing multiple identified drugs, 1 drug-centric sequence was generated for each *target-drug*. For example, for the sentence in Figure 4, the encoding scheme produced 2 labeled sequences: one with *lisinopril* as the *target-drug* and the other with *mirtazapine*. In each sequence, associated entities with the *target-drug* were labeled using a BIO scheme enhanced with their types. Hence, for the sequence generated with *lisinopril* as the *target-drug*, only *30 mg* and the first *QHS* were labeled using B and I tags, and other entities (eg, *15 mg*, *PO*, and the second *QHS*) were labeled as *O.*

We trained 2 separate models with the BiLSTM-CRF to jointly recognize (1) drug attributes and *drug−*{attributes} relations and (2) *ADE, reason*, and their corresponding relations (*drug−*{*ADE*, *reason*}). Similar to the *drug recognition model*, the input layer of these models takes word, character, and part-of-speech representations, with additional positional and semantic-tag embeddings. We used the positional embedding technique introduced in [82] to represent the positional distance from *target-drug* to each word in the input context. We used 3 different semantic tags, *target-drug, duplicate-target-drug,* and *nontarget-drugs*, to represent tokens of the current *target-drug*, other mentions of the same *target-drug*, and other drugs in the input context, respectively.

To handle intersentential relations, we provided adjacent sentences as an input context to the sentence containing the *target-drug*. We used training data to determine the optimal input context for the 2 models empirically. For the *drug−*{attributes} model, we determined the optimal context as the current sentence with the *target-drug* and the sentences preceding and following it. For the *drug−*{*ADE, reason*} model, the optimal context was the current sentence and the 4 sentences preceding and following it.

#### Contextual-Joint Model

We obtained domain-specific contextualized representations for input contexts by pretraining ELMo on MIMIC-III. These contextualized representations were used to augment the representations used in the input layers of the models in the *joint method*. With the augmented input representations, we trained (1) a *drug recognition* *model* and (2) 2 *drug-centric relation extraction* *models* (*drug−*{attributes} and *drug−*{*ADE, reason*}).

#### Positional-Joint Model

As the task involves extraction of drug-centric entities and relations, we used the position-attention mechanism to extract entities and relations jointly with respect to an entity of interest (*target-drug*).

Let 
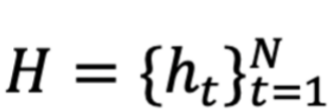
 represent the hidden representations of an input sequence obtained from the BiLSTM layer of the *contextual-joint model*. Positional representations 
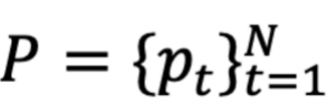
 were generated as follows:


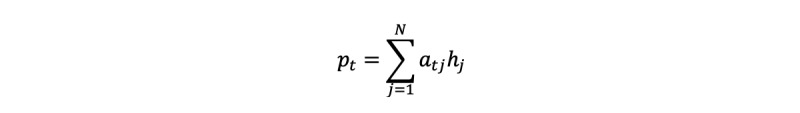



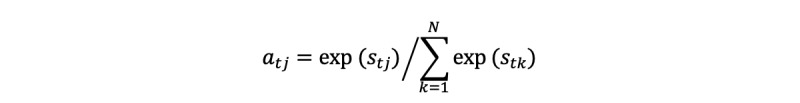



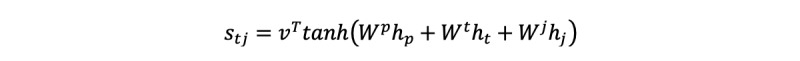


where *v, W^p^, W^t^, W^j^* are parameters to be learned, and *s_tj_* is the score obtained through additive attention. Position-attention computes dependencies among the hidden states: (1) *h_p_* at *target-drug* position *p*, (2) *h_j_* at *j^th^* token in the input sequence, and (3) *h_t_* at current token *t*. For each token *j, s_tj_* is computed by (1) comparing *h_p_* with *h_j_* and (2) comparing *h_t_* with *h_j_* The comparison of *h_p_* and *h_j_* helps to encode *target-drug* (positional) information, whereas the comparison of *h_t_* and *h_j_* is useful for matching sentence representations against itself (self-matching) to collect contextual information. *a_tj_* is the attention weight produced by the normalization of *s_tj_* and is used in computing the positional representation *p_t_* of the current token *t*. Finally, we concatenated this positional representation *p_t_* with its hidden representation *h_t_* to obtain *u_t_:*


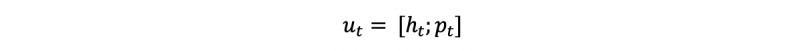


We trained the 2 *drug-centric relation extraction* *models* (*drug−*{attributes} and *drug−*{*ADE, reason*}) by feeding these concatenated representations to a CRF layer. During the test phase, we used the *drug recognition model* from the *contextual-joint* for predicting *drugs* and the trained *drug-centric relation extraction* *models* for predicting *drug−*{attributes} and *drug−*{*ADE, reason*} relations.

#### Knowledge-Joint Model

As introduced earlier, background knowledge and hidden relations beyond the contextual and positional information play a crucial role in extracting *drug−*{*ADE, reason*} relations. To address this, we propose the *knowledge-joint* model by enhancing the *positional-joint* model with knowledge embeddings created using the FAERS database.

Let 
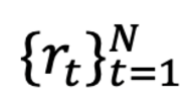
, 
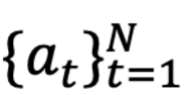
 denote representations of the input sequence tokens obtained from the *drug-reason* and *drug-adverse* knowledge embeddings, respectively. Let *l* and *m* be the beginning and end indices of *target-drug* in the input sequence. The *target-drug* *D_r_* and *D_a_,* corresponding to *drug-reason* and *drug-adverse* knowledge embeddings, were computed by averaging the representations of *target-drug* tokens:


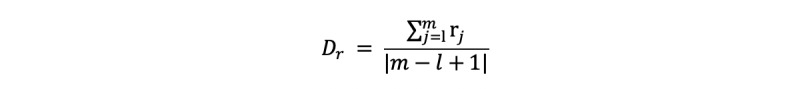



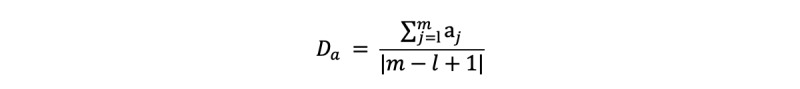


The *target-drug*–centric representations 
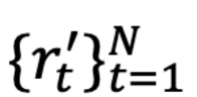
 and 
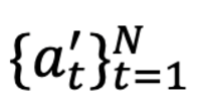
 were obtained by computing similarities between input sequence tokens and the *target-drug*:


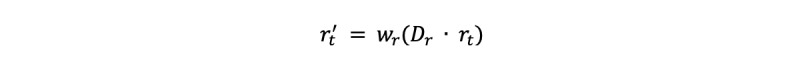



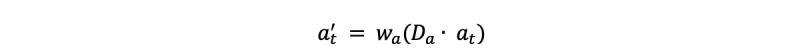


where *w_r_* and *w_a_* represent the scalar weights corresponding to *drug-reason,* and *drug-adverse* knowledge embeddings learned during training. Finally, for a token at position *t*, we concatenated its *target-drug*–centric similarities 
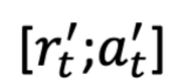
 with positional and hidden representations *u_t_* to produce *k_t_*:


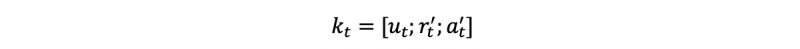


We trained a *drug-centric relation extraction* *model* (*drug−*{*ADE, reason*}) by feeding these concatenated representations to a CRF layer. During the test phase, we used the *drug recognition model* from the *contextual-joint* model for predicting *drugs* and the trained *drug−*{*ADE, reason*} *model* for predicting *drug−ADE* and *drug−reason* relations.

### Evaluation Metrics and Significance Tests

We evaluated the proposed system using the evaluation script released by the organizers of the n2c2 challenge to measure the lenient precision, recall, and F_1_ scores, explained as follows. For NER, a predicted entity is considered as a true-positive if its span overlaps with a gold annotation and is the correct entity type. For relation extraction, a predicted relation is considered as a true-positive if both entities in the relation are true-positives and the relation type matches the gold annotation. We also report statistical significance on these results with 50,000 shuffles and a significance level set to .05 by using a test script released by the n2c2 organizers based on the approximate randomization test [83].

In the following sections, we present the results of our system. The experimental settings used to achieve these results are provided in Multimedia Appendix 4.

## Results

### Named Entity Recognition

Table 3 presents the results for each proposed incremental approach for NER. Compared with the *joint method*, incorporating contextualized embeddings (*contextual-joint model*) improved the overall microaveraged F_1_ score by 0.3 percentage points. The improvement was mainly observed in recognizing *drugs* (0.6 points), with some improvements in recognizing *strength* and *reason*. Compared with the *contextual-joint model*, the *positional-joint model* improved the overall micro-F_1_ score by 0.2 points, with significant improvements observed in identifying *reason* (2.1 points) and *ADE* (6.8 points). Compared with the *positional-joint model*, the *knowledge-joint model* further improved the overall micro-F_1_ score by 0.1 points, with significant improvements observed in accurately determining *reason* (1.9 points) and *ADE* (1.7 points). Note that the overall improvement between the *positional-joint* and *knowledge-joint models* is relatively small due to the biased distribution of annotations, as *ADE* and *reason* together constitute less than 10% of the entities.

Significance tests showed that the improvements in micro-F_1_ score observed with each incremental approach are statistically significant with *P* values of .001, <.001, and <.001 for the *contextual-joint*, *positional-joint,* and *knowledge-joint* models, respectively. As the *contextual-joint* and *positional-joint models* share the same *drug recognition model*, we ignored drug predictions when performing significance tests. Similarly, the *positional-joint* and *knowledge-joint* models share the same *drug recognition model* and *drug−*{attributes} *model;* therefore, we considered only *ADE* and *reason* predictions when performing significance tests.

**Table 3:** Lenient precision, recall, and F1 score of the proposed approaches for named entity recognition.

Entity type	Joint			Contextual-joint			Positional-joint			Knowledge-joint		
	Precision	Recall	F_1_ score	Precision	Recall	F_1_ score	Precision	Recall	F_1_ score	Precision	Recall	F_1_ score
Drug	0.956	0.952	0.954	0.956	0.964	0.960	0.956	0.964	0.960	0.956	0.964	0.960
Strength	0.980	0.969	0.974	0.982	0.971	0.976	0.985	0.976	0.980	0.985	0.976	0.980
Form	0.974	0.942	0.958	0.975	0.939	0.957	0.972	0.943	0.958	0.972	0.943	0.958
Frequency	0.981	0.958	0.970	0.981	0.958	0.969	0.979	0.964	0.971	0.979	0.964	0.971
Route	0.964	0.942	0.953	0.962	0.943	0.952	0.950	0.949	0.949	0.950	0.949	0.949
Dosage	0.943	0.938	0.941	0.941	0.937	0.939	0.936	0.957	0.946	0.936	0.957	0.946
Duration	0.887	0.788	0.835	0.914	0.791	0.848	0.880	0.815	0.846	0.880	0.815	0.846
ADE^a^	0.649	0.358	0.462	0.643	0.346	0.450	0.660	0.426	0.518	0.589	0.490	0.535
Reason	0.757	0.611	0.676	0.747	0.636	0.687	0.747	0.672	0.708	0.753	0.702	0.727
Overall (micro)	0.948	0.912	0.929	0.947	0.917	0.932	0.943	0.926	0.934	0.941	0.930	0.935

^a^ADE: adverse drug event.

### Relation Extraction

Table 4 presents the results for each proposed incremental approach for relation extraction. Compared with the *joint method*, the *contextual-joint* model improved the overall micro-F1 score by 0.5 percentage points, with the majority of improvements observed in accurately recognizing *drug−strength, drug−frequency, drug−reason,* and *drug−dosage relations*. Compared with the *contextual-joint model*, the *positional-joint model* improved the F_1_ score by 0.4 points with significant improvements observed in determining *drug−ADE* (5.6 points) and *drug−reason* (2.9 points) relations. *The knowledge-joint model* further improved the overall F_1_ score by 0.1 points, with specific improvements in *drug−ADE* by 3.0 points and *drug−reason* by 1.7 points when compared with the *positional-joint model*. Similar to the NER significance results, significance testing for relation extraction showed that the improvements observed with each incremental approach are statistically significant with *P* values of <.001, <.001, and <.001 for the *contextual-joint*, *positional-joint*, and *knowledge-joint* models, respectively.

**Table 4:** Lenient precision, recall, and F1 score of the proposed approaches for relation extraction.

Relation type	Joint				Contextual-joint				Positional-joint				Knowledge-joint		
	Precision	Recall	F_1_ score	Precision		Recall	F_1_ score	Precision		Recall	F_1_ score	Precision		Recall	F_1_ score
Drug−strength	0.966	0.962	0.964	0.977		0.964	0.971	0.978		0.971	0.975	0.978		0.971	0.975
Drug−form	0.963	0.936	0.949	0.972		0.936	0.953	0.969		0.939	0.954	0.969		0.939	0.954
Drug−frequency	0.961	0.949	0.955	0.972		0.950	0.961	0.969		0.955	0.962	0.969		0.955	0.962
Drug−route	0.943	0.931	0.937	0.954		0.933	0.943	0.936		0.939	0.937	0.936		0.939	0.937
Drug−dosage	0.921	0.928	0.924	0.933		0.931	0.932	0.925		0.950	0.937	0.925		0.950	0.937
Drug−duration	0.814	0.718	0.763	0.880		0.723	0.794	0.823		0.739	0.779	0.823		0.739	0.779
Drug−ADE^a^	0.590	0.322	0.417	0.592		0.307	0.404	0.590		0.377	0.460	0.544		0.446	0.490
Drug−reason	0.682	0.526	0.594	0.676		0.546	0.604	0.680		0.593	0.633	0.673		0.628	0.650
Overall (micro)	0.912	0.859	0.885	0.920		0.862	0.890	0.912		0.877	0.894	0.906		0.884	0.895

^a^ADE: adverse drug event.

## Discussion

### Principal Findings

Contextualized representations (*contextual-joint*) are effective in differentiating between words and abbreviations that could have multiple meanings. For example, *ensure* and *contrast* can be understood as either a *drug* (“Ensure: 1 can PO three times daily” and “contrast-induced nephropathy”) or a verb, and terms such as *blood* could either refer to a drug (“transfused 1 unit of blood”), that is, substance given to a patient, a test for the drug (“blood alcohol concentration”), or a natural occurring substance in the body (“blood pressure”). Additionally, abbreviations such as *PE* (physical examination versus pulmonary embolism) and *pcp* (primary care physician versus pneumocystis pneumonia) can have multiple expansions. In all the examples above, the *contextual-joint* correctly identifies these entities.

**Figure 5:**
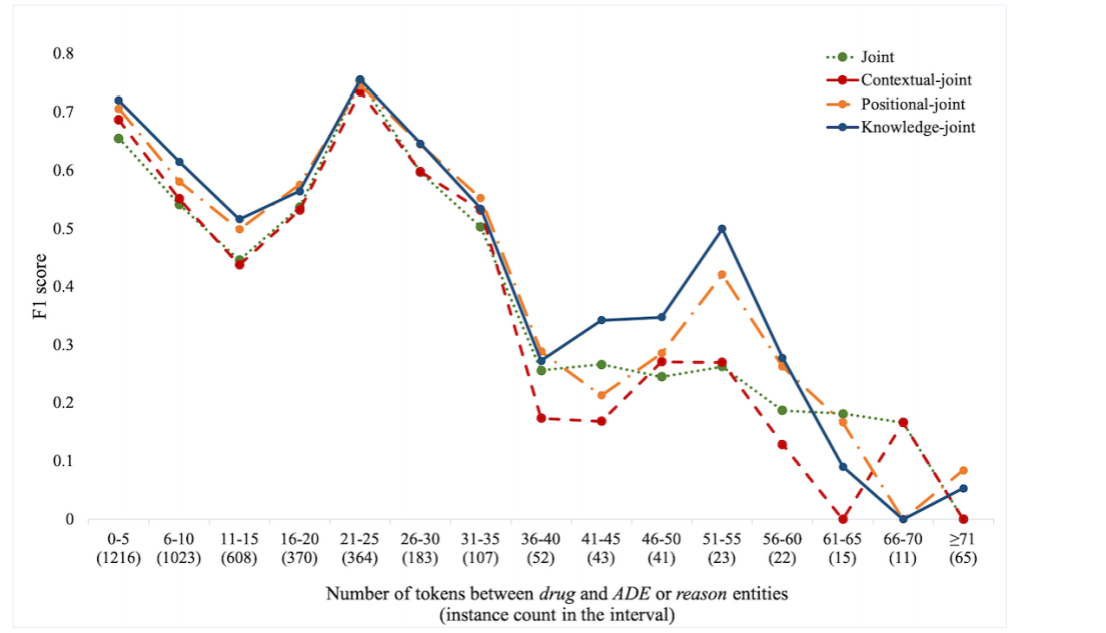
F1 scores of approaches with increasing distance between entities for relation extraction. ADE: adverse drug event.

One prevailing challenge in ADE extraction is the presence of long-distance or intersentential relations. As shown in Table 2, a significant portion of *drug*−{*ADE, reason*} in the data set is intersentential (23% of *drug*−*ADE* and 31.7% of *drug*−*reason*). These relations typically span long distances, making them more difficult to capture. To study the effectiveness of the proposed approaches over long-distance relations, we calculated the F_1_ scores on *drug*−{*ADE, reason*} with an increasing number of tokens between entities. As shown in Figure 5, we find that the positional*-joint* model performs significantly better than the *contextual-joint* model with increasing distance between entities, suggesting that the *positional-joint* can effectively model long-distance relations.

Incorporating knowledge embeddings learned on the FAERS improved *drug*−{*ADE, reason*} relation extraction, especially in the case of long-distance relations or when contextual clues are insufficient. As shown in Figure 5, *the knowledge-joint* model further improved on the *positional-joint* model at all distances. The *knowledge-joint* model was also useful in cases of insufficient or ambiguous context in extracting the correct relation. For example, in the phrase “Wellbutrin - nausea and vomiting,” the relation is indicated only by an uninformative hyphen, with no contextual clues to indicate the type of relation. Similarly, in “Patient had history of depression and was on elavil previously,” it is unclear whether the *history of depression* was previously treated by *drug*−*reason* or caused by *drug*−*ADE* of the drug *elavil*. Furthermore, the *knowledge-joint* also helped to extract correct relations when multiple drugs and candidate *ADEs* and *reasons* are discussed in a given context. For example, in “Upon arrival, she was hypertensive and had a fever. She was given Tylenol*,*” based on sentence construction, 2 candidate *reasons* (*hypertensive* and *fever*) may be associated with the *drug* *Tylenol*. Knowledge is required to infer that of the two, only *fever* is related to *Tylenol*.

### Error Analysis

We investigated the most common error categories by entity and relation type and present these in Table 5. Most of the errors in recognizing *drug*s were due to abbreviations, misspellings, generic terms, or linguistic shorthand. For *strength* and *dosage*, these entities were often mislabeled as each other—both are often numeric quantities and used in similar contexts. For *duration* and *frequency*, most of the errors resulted from these entities being expressed in colloquial language.

Intersentential relations remain a major category of false-negative errors for all relations despite improvements from the position-attention mechanism. For *drug−*{attributes}, these errors were likely due to an insufficient number of such examples in the training data (approximately 4%). In addition to errors from intersentential relations, other important categories for false-negative *drug−*{*ADE, reason*} include (1) *ADE* or *reasons* expressed in generic terms, (2) *reasons* such as procedures and activities (eg, *angioplasty/stenting*) that occur infrequently in the training set*,* and (3) *ADE* or *reasons* expressed as abbreviations that are nonstandard or ambiguous*.*

False-positive errors in *drug−*{*ADE*, *reason*} mainly fall into 2 categories. In the first, one of the entities participating in the relation is negated, hypothetical, or conditional, such as when a drug is withheld to avoid an anticipated ADE (eg, contraindications). In the second, the same concept (*drug*, *ADE*, or *reason*) is mentioned multiple times in the same context, and the system associated the relation to one mention whereas the ground truth to the other. To add further complexity, these mentions may be synonyms, for example, “the pain medications (morphine, vicodin, codeine) worsened your mental status and made you delirious.” With multiple possible *drug*−*ADE* relations, some combinations were not captured in the ground truth, resulting in false-positives that may not be true errors.

**Table 5:** Error analysis on our best-performing model (knowledge-joint).

Entity/relation, Error category		Text^a^	Explanation
**Drug**			
	Abbreviation	Hyponatremia due to *HCTZ*^b^	HCTZ—abbreviated drug
	Misspelled words	30 units of Lantus in addition to *humalong*	Humalog is incorrectly written as humalong
	Short forms	She was given *vanco*	Vancomycin is expressed in shorthand
	Generic phrase	He was advised to not take any of his *blood pressure medications*	Antihypertensives are expressed through generic terms
**Strength**			
	Contextual ambiguity	Patient received *1 unit* of *blood*	*Strength* (*1 unit*) wrongly predicted as *dosage*; usually, the *unit* token is associated with *dosage*
**Duration**			
	Colloquial language	Only take *Hydroxyzine* * as long as your rash is itching*	*Duration* is expressed colloquially
**Drug** **−** **strength**			
	Intersentential	Continued *Carvedilol*. INR^c^ initially slightly supratherapeutic, but then his home regimen of *4mg* alternating with *2mg* daily was started	Intersentential relation between carvedilol and 4 mg
**Drug** **−** **ADE^d^** **; Drug** **−** **reason**			
	Intersentential	He underwent *coronary artery bypass* x5, please see operative report for further details. He was transferred to the CSRU^e^ on *Neo* with IABP^f^	Intersentential relation between neo and coronary artery bypass graft
	Generic terms	Start a baby *aspirin* every day to *protect the heart*	*Reason* is expressed in generic terms
	Abbreviation	*Detrol* was discontinued on suspicion that it might contribute to *AMS*	*AMS* has multiple possible expansions
	Procedure	*Angioplasty* of the left tibial artery; had been on *Plavix* prior to NSTEMI^g^	Procedure angioplasty is annotated as *reason*
	Contraindication	Avoiding *NSAIDs*^h^ to prevent *gastrointestinal bleed*	*Drug* was not given to this patient
	Negated	*Heparin*-induced *thrombocytopenia* negative	*ADE* thrombocytopenia is negated

^a^Italics indicate text that contributes to the specified error category.

^b^HCTZ: hydrochlorothiazide.

^c^INR: international normalized ratio.

^d^ADE: adverse drug event.

^e^CSRU: cardiac surgery recovery unit.

^f^IABP: intra-aortic balloon pump.

^g^NSTEMI: non–ST-elevation myocardial infarction.

^h^NSAIDs: nonsteroidal anti-inflammatory drugs.

### Document-Level Analysis

From an end user perspective, the core information needed for patient care purposes is a patient-level summary of these relations, which is a unique set of extracted relations after normalization. To evaluate our system for this purpose, we measured *drug−ADE* and *drug−reason* F_1_ scores by considering unique pairs of relation mentions at the document level, presented in Table 6. We observed scores at the document level to be 1 to 2 percentage points higher than the instance level.

**Table 6:** Document-level analysis for drug−reason and drug−adverse drug event relations.

Model	Drug−reason						Drug−ADE^a^					
	Instance level			Document level			Instance level			Document level		
	Precision	Recall	F_1_ score	Precision	Recall	F_1_ score	Precision	Recall	F_1_ score	Precision	Recall	F_1_ score
Joint	0.682	0.526	0.594	0.691	0.542	0.607	0.590	0.322	0.417	0.631	0.322	0.426
Contextual-joint	0.675	0.546	0.604	0.685	0.560	0.616	0.592	0.307	0.404	0.630	0.308	0.414
Position-joint	0.680	0.593	0.633	0.692	0.611	0.649	0.590	0.376	0.460	0.647	0.384	0.482
Knowledge-joint	0.673	0.628	0.650	0.687	0.647	0.666	0.544	0.446	0.490	0.579	0.444	0.503

^a^ADE: adverse drug event.

### Comparison With Previous Work

For NER, the state-of-the-art system [38] used an ensemble (committee) of 3 different methods: CRF, BiLSTM-CRF, and joint approach. They showed that the BiLSTM-CRF is the best among the single models. Thus, we compare our best model (*knowledge-joint*) with their best-performing single model and committee approach, as shown in Table 7. Overall, *the knowledge-joint* model outperformed the single model by 0.2 percentage points and achieved similar micro-F_1_ to the committee approach. Notably, *the knowledge-joint* model significantly outperformed the committee approach in recognizing the crucial *ADE* (0.5 points) and *reason* (5.2 points) entities.

For relation extraction, the state-of-the-art system used the committee approach for NER, convolutional neural network – recurrent neural network (CNN-RNN) for relation classification, and postprocessing rules. Although postprocessing rules are commonly used in competitions, they often do not generalize across data sets and therefore are of limited interest in this research. As shown in Table 7, *the knowledge-joint model* outperformed the state-of-the-art approach, both with (0.4 points) and without rules (1.6 points). Notably, *the knowledge-joint* model achieved the best results and outperformed the state-of-the-art in recognizing the most crucial and difficult to extract relations: *drug−reason* (7.1 points) and *drug−ADE* (1.4 points).

**Table 7:** The lenient F1 scores for named entity recognition of single and state-of-the-art ensemble models compared with our best model. The lenient F1 scores for relation extraction of state-of-the-art ensemble models with and without rules, compared with our best model.

NER^a^					Relation extraction			
Entity type	BiLSTM-CRF^b^ [38]	Committee [38]	Knowledge-joint	Relation type		Committee + CNN-RNN^c^ [38]	Committee + CNN-RNN + Rules [38]	Knowledge-joint
Drug	0.955	0.956	0.960	N/A^d^		N/A	N/A	N/A
Strength	0.982	0.983	0.980	Drug−strength		0.964	0.972	0.975
Form	0.958	0.958	0.958	Drug−form		0.940	0.952	0.954
Frequency	0.974	0.975	0.971	Drug−frequency		0.941	0.958	0.962
Route	0.956	0.956	0.949	Drug−route		0.930	0.942	0.937
Dosage	0.943	0.948	0.946	Drug−dosage		0.923	0.935	0.937
Duration	0.856	0.862	0.846	Drug−duration		0.740	0.786	0.779
ADE^e^	0.422	0.530	0.535	Drug−ADE		0.475	0.476	0.490
Reason	0.680	0.675	0.727	Drug−reason		0.572	0.579	0.650
Overall (micro)	0.933	0.935	0.935	Overall (micro)		0.879	0.891	0.895

^a^NER: named entity recognition.

^b^BiLSTM-CRF: bidirectional long short-term memory–conditional random field.

^c^CNN-RNN: convolutional neural network–recurrent neural network.

^d^Not applicable.

^e^ADE: adverse drug event.

### Limitations and Future Work

We acknowledge several limitations of this study. First, these results are specific to the n2c2 data set, which contains only intensive care unit (ICU) discharge summaries from a single health care organization. Ground truth generation and evaluation on a more diverse data set is needed to better understand the effectiveness of these proposed approaches. Second, we observed some annotation errors in the ground truth, likely due to the complex nature of the task. Further investigation is needed to quantify the prevalence of such errors and their impact on the results.

Despite achieving state-of-the-art results, the proposed system still has room for improvement, specifically in recognizing intersentential *drug*−{*ADE*, *reason*} relations. To further improve ADE extraction, we plan to explore the following research areas:

1. Although we incorporated knowledge graph embeddings, other advanced methods that use higher-order proximity and role-preserving network embedding techniques have shown promising results in the general domain. We plan to explore methods such as Edge Label Aware Network Embedding [84] rather than training separate graph embeddings for *drug−{ADE, reason}* relations.
2. The field of contextual embeddings has evolved quickly along with the release of newer language representation models trained on clinical text. We plan to explore BERT [78,85], which utilizes a transformer network to pretrain a language model for extracting better contextual word embeddings.
3. To address some of the findings from the error analysis, we plan to leverage our clinical abbreviation expansion components [86] to help resolve ambiguous mentions and also incorporate assertion recognition [26] to capture the belief state of the physician on a concept (negated, hypothetical, conditional).
4. As mentioned earlier, the proposed models performed poorly on intersentential relation extraction. To address this, we plan to explore N-ary relation extraction for cross-sentence relation extraction using graph long short-term memory networks [87].

### Conclusions

We presented a system for extracting drug-centric concepts and relations that outperformed current state-of-the-art results. Experimental results showed that contextualized embeddings, position-attention mechanisms, and knowledge embeddings effectively improve deep learning-based concepts and relation extraction. Specifically, we showed the effectiveness of a position-attention mechanism in extracting long-distance relations and knowledge embeddings from the FAERS in recognizing relations where contextual clues are insufficient.

## Appendix

Multimedia Appendix 1 Sentence segmentation and tokenization.

Multimedia Appendix 2 Embeddings from Language Models contextualized embeddings and large-scale information network embedding graph embeddings.

Multimedia Appendix 3 Detailed explanation of bidirectional long short-term memory–conditional random fields and input embeddings.

Multimedia Appendix 4 Experimental settings used in the proposed system.

## Abbreviations

ADE: adverse drug event
AEOLUS: adverse event open learning through universal standardization
BERT: Bidirectional Enconder Representations from Transformers
BiLSTM-CRF: bidirectional, long short-term memory–conditional random fields
BIO: beginning, inside, and outside
CNN: convolutional neural network
CRF: conditional random field
CUI: concept unique identifier
EHR: electronic health record
ELMo: Embeddings from Language Models
FAERS: Food and Drug Administration Adverse Event Reporting System
LINE: large-scale information network embedding
MADE 1.0: Medication and Adverse Drug Events from Electronic Health Records
MIMIC-III: Medical Information Mart for Intensive Care III
n2c2: 2018 National NLP Clinical Challenges
NER: named entity recognition
NLP: natural language processing
SBD: sentence boundary detection
UMLS: Unified Medical Language System
