# Supplementary material for: Extraction of Information Related to Drug Safety Surveillance From Electronic Health Record Notes: Joint Modeling of Entities and Relations Using Knowledge-Aware Neural Attentive Models
Source: JMIR Med Inform. 2020 Jul 10;8(7):e18417. doi: 10.2196/18417 (PMC7382020; doi:10.2196/18417)
Supplement: Multimedia Appendix 1 [file medinform_v8i7e18417_app1.pdf]

SENTENCE SEGMENTATION AND TOKENIZATION

We extended Spacy[1] with custom rules that can capture the inherent writing style of clinical narratives and generate better low-level features such as sentences and tokens. We performed three steps to generate these features namely: *1) Pseudo paragraph generation 2) Tokenization 3) Sentence Segmentation.*

Table 1. Custom affix rules for tokenization

Name	Description	Additional affix rules	Text	Tokens
Prefixes	A regex-based function for identifying token prefixes	'[**', '**]', (\d+/\d+ | \d+\,\d+ | \d*.\d+ | \d+. | \d+), '-', '.', 'O2', 'o2'	[**2012	[**, 2012
			2.3units	2.3, units
Infixes	A regex-based function for identifying the infixes in a token	'(', '+', '->', '/', '-', ':'	50+units lisin/hctz	50, +, units lisin, /, hctz
Suffixes	A regex-based function for identifying token suffixes	'[**', '**]', 'mg', 'prn', 'qhs', 'hrs', 'O2', 'o2', '(s)', '-', ':', 'NC', 'SQ', 'PRBC', 'QAM', 'QPM', 'PM', 'nc', 'MWF', 'QD', 'RBCs'	20mg 8hrs	20, mg 8, hrs

Pseudo paragraph generation

As a first step, a clinical narrative is segmented into multiple *pseudo paragraphs* (a pseudo paragraph is a continuous group of multiple sentences). A *newline character* serves as a good separator for splitting paragraphs but because of spurious newline characters introduced due to automatic line wrapping[2] in EHR systems, we applied the following rules to selectively retain/replace the newline character.

1. A newline character is *retained* if either:
    - the next line starts with a bullet point pattern ("\d+\.")
    - the current and next line start with a medication.
        - This rule helps in accurately segmenting imported medication sections in EHRs. A list of *medication strings* is compiled from UMLS and a simple lookup is performed to determine whether a sentence starts with medication or not.
2. A newline character is *replaced* by a space if either:
    - the total number of characters in the current sentence line range between *70 and 85 characters*. This helps in addressing issues with automatic line wrapping as it happens around this interval in this dataset.

- the next line starts with a lower-case character.

3. Next, each of these obtained *pseudo paragraphs* are fed to customized Spacy to identify the token and sentence boundaries.

Tokenization

Spacy uses *white space* to segment text into multiple tokens and iteratively uses a pre-compiled list of *affixes (prefixes, suffixes and infixes)* to further segment a token. Spacy allows users to customize these *affixes*, thus, we empirically added several *affix rules* using the provided dataset. These rules with corresponding illustrations are provided in Table 1. Furthermore, Spacy allows users to define *special cases* such as when to leave tokens containing periods intact (abbreviations like "D.M.") or when to split them. We provided a special case with list of all clinical abbreviations obtained from UMLS to prevent these tokens from *incorrectly splitting into multiple tokens.*

Sentence segmentation

Table 2. Token level rules for obtaining better sentence boundaries

Name	Description	Rule
Unfinished parenthesis	Sentence can't start on tokens present within unfinished parenthesis	Any token between:<br>a) '[**', '**]'<br>b) '(' , ')'<br>c) '{' , '}'<br>d) '[' , ']'
Punctuation marks	Sentence can't start on punctuation marks	[':' , '/' , '*' , ',']
Dosage instruction	Sentence can't start in the middle of a dosage instruction	Tokens within the pattern:<br>NUM [SPACE]*<br>[mg/mcg/unit]<br>[tablet/capsule]

Spacy features a fast and accurate *syntactic dependency parser* which also powers the *sentence boundary detection (SBD)* i.e., the SBD relies on syntactic dependency parse. However, Spacy allows users to customize several token level parameters for customizing sentence segmentation, of which *is_sent_start* is used to configure whether a sentence can be started on a particular token text or a token text that matches a particular pattern. Because of de-identified tokens and templates in the clinical narratives, Spacy tends to aggressively split at these positions. To address this, we added several exception rules using *is_sent_start* parameter which are illustrated in Table 2.

RESULTS

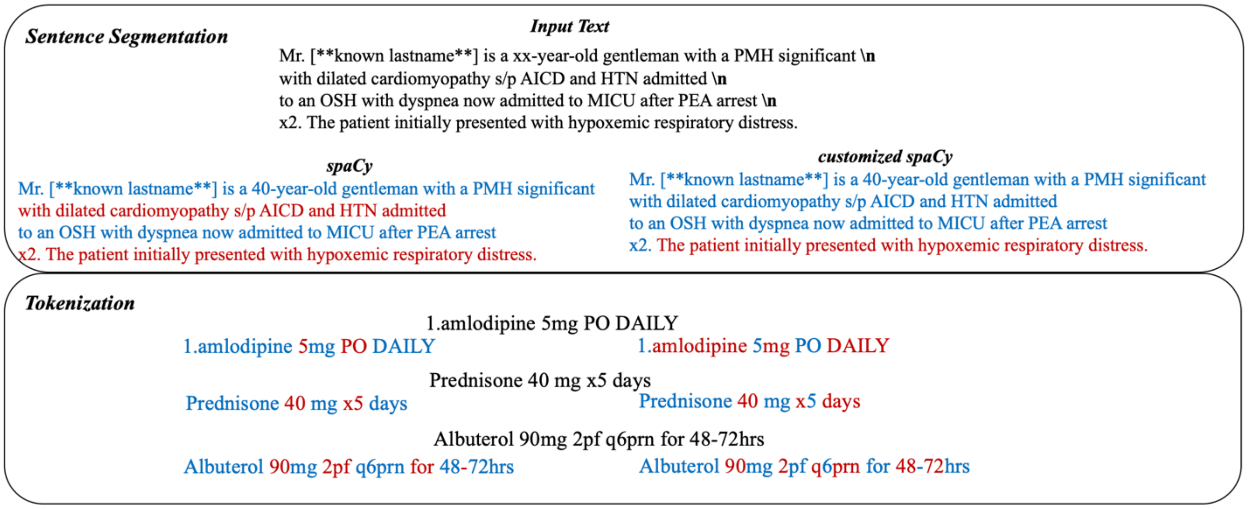

Figure 1. Tokenization and Sentence Segmentation differences between Default and Customized Spacy. Two consecutive tokens and sentences are indicated using alternative colors.

Figure 1 provides an illustration of *sentence segmentation and tokenization results* obtained using default and customized Spacy. As shown in Figure 1, the sentences obtained from default Spacy were incorrectly segmented at newline characters and the correct sentence boundary (at '*x2*') was not recognized. With the help of pseudo paragraph generation, the customized Spacy replaced newline characters with white space and hence was able to accurately detect sentence boundaries. Furthermore, due to the absence of white space, default Spacy didn't recognize the correct token boundaries (e.g, '*1.amlodipine*', '*q6prn*'). However, customized Spacy generated accurate tokens with the help of additional tokenization rules.

REFERENCES

1. Honnibal M, Montani I. spaCy2: Natural language understanding with bloom embeddings, convolutional neural networks and incremental parsing. Features. 2017.
2. Zweigenbaum P, Grouin C, Lavergne T. Supervised classification of end-of-lines in clinical text with no manual annotation. Proc Fifth Work Build Eval Resour Biomed Text Min 2016. p. 80–88.
