# Supplementary material for: Extraction of Information Related to Drug Safety Surveillance From Electronic Health Record Notes: Joint Modeling of Entities and Relations Using Knowledge-Aware Neural Attentive Models
Source: JMIR Med Inform. 2020 Jul 10;8(7):e18417. doi: 10.2196/18417 (PMC7382020; doi:10.2196/18417)
Supplement: Multimedia Appendix 2 [file medinform_v8i7e18417_app2.pdf]

EMBEDDINGS FROM LANGUAGE MODELS (ELMO)

ELMo[1] is one of the deep contextualized word representation models. First, ELMo trains a bi-directional language model (BiLM) on a large corpus by feeding character representations through a multi-layer Bi-LSTM network. Then, ELMo learns the task specific contextual representations of a word through a linear combination of the intermediate layer representations in BiLM.

Table 1: Hyperparameter values used for retraining ELMo model

**Hyperparameter**		**Value**
Char-CNN	Character embedding size	16
	Filters (width, number)	[(1, 32), (2, 32), (3, 64), (4, 128), (5, 256), (6,512), (7, 1024)]
	Maximum characters per token	50
	Number of highway layers	2
	Activation function	ReLU
Bi-LSTM	Hidden layer size	4096
	Number of Bi-LSTM layers	2
	Final projection dimension	512

Formally, given a sequence of tokens $(T_1, \ldots, T_N)$, the forward language model computes the probability of a token T_t given its history (T_1, …, T_{t-1}) through chain rule:

$$p(T_{1,}T_2 \ldots T_N) = \prod_{t=1}^{N} p(T_t | T_1, T_2 \ldots T_{t-1})$$

and similarly, a backward language model computes the probability of a token given its future tokens $(T_{t+1}, T_{t+2} \ldots T_N)$:

$$p(T_{1,}T_2 \ldots T_N) = \prod_{t=1}^{N} p(T_t | T_{t+1}, T_{t+2} \ldots T_N)$$

At each position t, a context-independent token-representation x_t^{LM} is obtained using a convolutional neural network on character representations. These representations are passed through L layers of forward and backward LSTMs. These layers output a context-dependent representation $\overrightarrow{h_{t,J}^{LM}}$ and $\overleftarrow{h_{t,J}^{LM}}$ where $j = 1,2 \ldots.L$ and the top forward and backward LSTM layer outputs $\overrightarrow{h_{t,L}^{LM}}$ and $\overleftarrow{h_{t,L}^{LM}}$ are fed into a SoftMax layer. The objective function is to maximize the likelihood of forward and backward language models:

$$\sum_{t=1}^{N}(\log p(T_t|T_1, T_2 \dots T_{k-1}; \Theta_x, \overrightarrow{\Theta_{LSTM}}, \Theta_s) + \log p(T_t|T_{t+1}, T_{t+2} \dots T_N; \Theta_x, \overleftarrow{\Theta_{LSTM}}, \Theta_s)$$

where (Θ_x), (Θ_s),$(\overrightarrow{\Theta_{LSTM}})$ and $(\overleftarrow{\Theta_{LSTM}})$ are token representation, SoftMax layer, forward and backward LSTM parameters respectively.

We trained ELMo model on MIMIC-III corpus by using the default parameters as listed in Table 1 for 10 epochs with a dropout value of 0.1.

LARGE-SCALE INFORMATION NETWORK EMBEDDING (LINE)

LINE[2] is one the network embedding models that suits arbitrary types of information networks and scales to large weighted graphs by using alias table method[3] to draw samples according to edge weights. LINE preserves both the explicit (first order) and implicit (second order) relations in learning the network embeddings of nodes, ensuring two nodes directly linked or sharing "context" to have similar embeddings.

Formally, let $G' = (\boldsymbol{d}, \boldsymbol{o}, \boldsymbol{e})$ be a weighted bipartite network, where $d = \{d_1, d_2, \dots, d_L\}$ and $o = \{o_1, o_2, \dots, o_M\}$ denote the set of L drug tokens and M outcome tokens, and $\boldsymbol{e} \subseteq \boldsymbol{d} \times \boldsymbol{o}$ defines the inter-set edges, each edge $e_{lm} \in \boldsymbol{e}$ carries a non-negative weight w_{lm}. The joint probability $P(d_l, o_m)$ between a drug token d_l and an outcome token o_m token is defined as:

$$P(d_l, o_m) = \frac{w_{lm}}{\sum_{e_{ij} \in e} w_{ij}}$$

The first-order proximity is the observed pairwise proximity between a drug token d_l and outcome token o_m. To preserve the first-order proximity, a sigmoid function is used to transform the interaction between a drug token d_l and outcome token o_m to the probability space:

$$p_1(d_l, o_m) = \frac{1}{1 + exp\,(-\vec{d}_l^T . \vec{o}_m)}$$

where $\vec{d}_l \in R^d$ and $\vec{o}_m \in R^d$ are first order embedding vectors of nodes d_l and o_m respectively.

The second-order proximity assumes that vertices of one type sharing many connections to vertices of other type (i.e., vertices which share similar “contexts”) are similar to each other. Therefore, each vertex plays two roles: the vertex itself and a “context” of other vertices. For a given vertex d_l, $\vec{d_l}$ and $\overrightarrow{d_l}'$ denotes its representation when it is treated as a vertex and “context” of other vertices respectively. Without loss of generality, we transformed the network to a directed graph by replacing each undirected edge with two directed edges with opposite directions and equal weights. For each directed edge, the second order proximity is modeled by the conditional probability of the context node o_m being generated by a node d_l is given as:

$$p_2(o_m|d_l) = \frac{\exp\left(\overrightarrow{o_m}'^{T}.\vec{d_l}\right)}{\sum_{k=1}^{|M|}\exp\left(\overrightarrow{o_k}'^{T}.\vec{d_l}\right)}$$

By minimizing the KL-divergence KL_1 and KL_2 of these two distributions p_1 and p_2 separately, the low-dimensional representations of nodes which are able to preserve both the first and second order proximities are obtained.

$$KL_1 = -\sum_{e_{ij}\in E} w_{ij} log\ \ p_1(d_i, o_j)$$

$$KL_2 = -\sum_{e_{ij}\in E} w_{ij} log\ \ p_2(o_j|d_i)$$

The final representation of a node is obtained by concatenating the first and second order embeddings.

We used the default parameters provided in [2] to train our models.

REFERENCES

1 Peters M, Neumann M, Iyyer M, *et al.* Deep Contextualized Word Representations. 2018. 2227–37.

2 Tang J, Qu M, Wang M, *et al.* LINE: Large-scale information network embedding. In: *WWW 2015 - Proceedings of the 24th International Conference on World Wide Web*. 2015. 1067–77.

3 Li AQ, Ahmed A, Ravi S, *et al.* Reducing the sampling complexity of topic models. In: *Proceedings of the ACM SIGKDD International Conference on Knowledge Discovery and Data Mining*. 2014. 891–900.
