# Supplementary material for: Extraction of Information Related to Drug Safety Surveillance From Electronic Health Record Notes: Joint Modeling of Entities and Relations Using Knowledge-Aware Neural Attentive Models
Source: JMIR Med Inform. 2020 Jul 10;8(7):e18417. doi: 10.2196/18417 (PMC7382020; doi:10.2196/18417)
Supplement: Multimedia Appendix 3 [file medinform_v8i7e18417_app3.pdf]

BILSTM-CRF

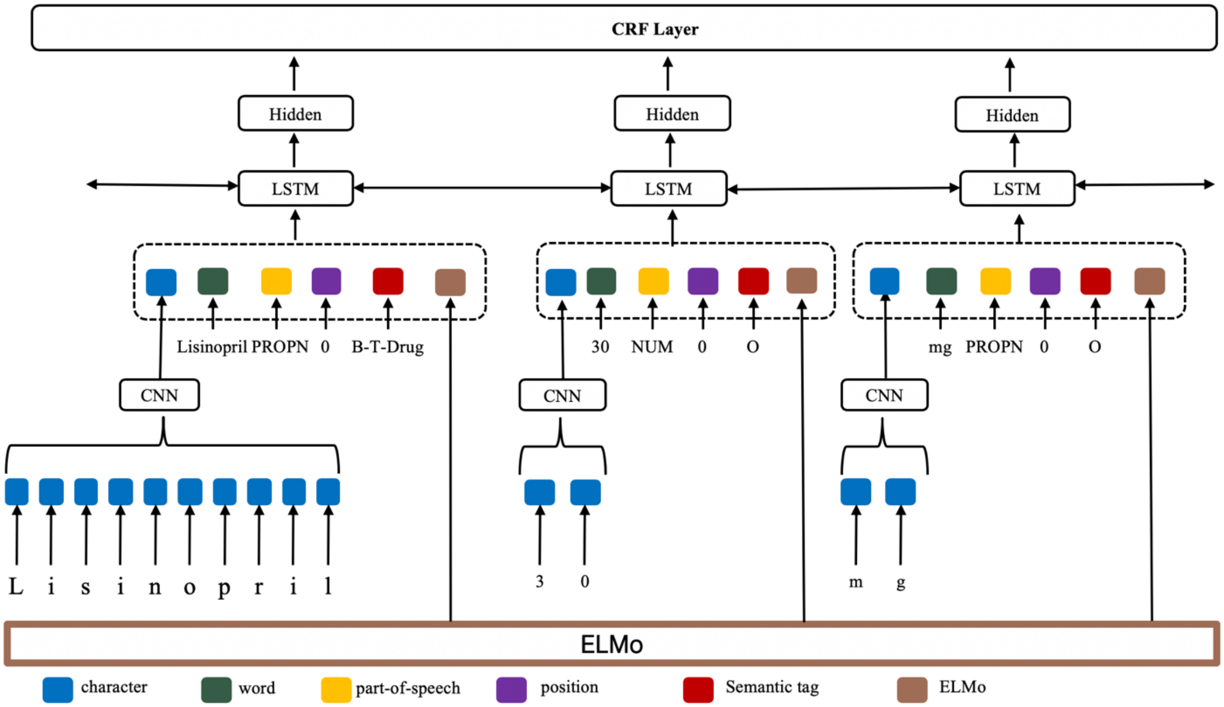

Figure 1. Overview of all embedding types used in our system.

With recent advances in NLP research, several neural network architectures have been successfully applied to entity and relation extraction tasks. Specifically, Long Short-Term Memory[1] based architectures have proven to be effective. Long short-term memory (LSTM) a type of recurrent neural network (RNN) that model interdependencies in sequential data and addresses the so-called vanishing or exploding gradients problem[2] of vanilla RNNs by using adaptive gating mechanism. Unidirectional LSTMs do not utilize the future contextual information. Bidirectional LSTMs[3,4] addresses this by using two independent LSTMs (forward and backward) in which one processes the input sequence in the forward direction, while the other processes the input in the reverse direction. Although Bidirectional LSTM networks have the ability to capture long distance interdependencies, previous research[5,6] suggests additionally capturing the correlations between adjacent labels by augmenting conditional random fields (CRF) can help in sequence labeling problems.

Formally, given an input sequence with tokens $(T_1, T_2, \dots, T_N)$, we generate input sequence representation $x = (x_1, x_2, \dots, x_N)$ where N is the sequence length, then the LSTM hidden state at timestep t is computed by:

$$i_t = \sigma(W^i x_t + U^i h_{t-1} + b^i)$$

$$
\begin{aligned}
f_t &= \sigma(W^f x_t + U^f h_{t-1} + b^f) \\
o_t &= \sigma(W^o x_t + U^o h_{t-1} + b^o) \\
g_t &= tanh(W^g x_t + U^g h_{t-1} + b^g) \\
c_t &= f_t \otimes c_{t-1} + i_t \otimes g_t \\
h_t &= o_t \otimes \tanh(c_t)
\end{aligned} \tag{1}
$$

where $\sigma(.)$ and $\tanh(.)$ are the element-wise sigmoid and hyperbolic tangent functions, $\otimes$ is the element-wise multiplication operator, and i_t, f_t, and o_t are the input, forget, and output gates. Lastly, h_{t-1} and $c_{t-1}$are the hidden state and memory cell of previous timestep respectively.

The forward LSTM computes the forward hidden states ($\overrightarrow{h_1}$, $\overrightarrow{h_2}$,…, $\overrightarrow{h_N}$) while the backward LSTM computes backward hidden states ($\overleftarrow{h_1}$, $\overleftarrow{h_2}$,…, $\overleftarrow{h_N}$). Then for each timestep t, the hidden state of the Bi-LSTM is generated by concatenating $\overrightarrow{h_t}$ and $\overleftarrow{h_t}$ as in:

$$h_t = (\overrightarrow{h_t}, \overleftarrow{h_t}) \tag{2}$$

Given an observation sequence h= $(h_1, h_2, \dots, h_N)$ (outputs from Bi-directional LSTM), CRF jointly models the probability of the entire sequence of labels $L = (l_1, l_2, \dots, l_N)$ by using the discriminative probability to l_t given h_t and the transition probability between adjacent labels. Using linear-chain CRF model, then the conditional probability of the output sequence, given the input hidden state sequence can be written as:

$$P(L|h; W, b) = \frac{\prod_{t=1}^{N} \psi(l_{t-1}, l_t, h)}{\sum_{l' \in \varphi(H)} \prod_{t=1}^{N} \psi\left(l'_{t-1}, l, h\right)},$$

Where $\psi\left(l', l, h\right) = \exp\left(W_{l',l}^T h_i + b_{l',l}\right)$ is the potential function and $W_{l',l}^T$ and $b_{l',l}$ are weight vector and bias corresponding to the label pair label pair (l', l).

We use maximum conditional likelihood estimate and gradient ascent to train the CRF layer. For a training dataset with M sequences $\{(h^i, L^i)_{i=1}^M\}$, the final log-likelihood is:

$$L(W, b) = \sum_i log\ P(L^i | h^i; W, b)$$

For the decoding phase, Viterbi algorithm is used to generate the optimal label sequence L^*

$$L^* = \underset{L \in \varphi(h)}{\operatorname{argmax}}\, p(L|h; W, b)$$

INPUT REPRESENTATIONS

The syntactic and semantic information in a sentence are fed in the form of various embeddings into neural networks. Figure 1 depicts all the embedding types that are used in our current system.

Let $S = \{T_1, T_2 \dots T_N\}$ be a sentence with N tokens, for each token T_t we provide input representations to BiLSTM-CRF in the form of embeddings obtained using six different types of features, namely, word (w_t), part-of-speech (pos_t), ElMo (e_t), positional (p_t), character (c_t) and semantic tag (s_t). The overall representation of each token is obtained by concatenating these features.

$$x_t = [w_t, c_t, pos_t, e_t, p_t, s_t]$$

Word Embeddings

The word embedding (w_t) of a given token is obtained by performing lookup operation on pretrained embeddings created using MIMIC-III corpora. For tokens without corresponding pre-trained embeddings, their word embeddings are initialized with uniformly distributed random values.

Character Embeddings

The main advantages with using character embeddings is their ability a) to capture shape and morphological features of a token and b) to generate representations even for out-of-vocabulary words as we can embed any word through its character representations. We followed the convolutional neural network architecture proposed in [7] for generating character embeddings without using any highway layers (highway layers did not improve overall performance of our system). For a token x_t, the token-level character embedding c_t is obtained by performing convolutions on its character representations with fixed filter size of 10 and varying kernel widths (1,2,3,4,5) followed by a max pooling layer. We preserved the *case* of all characters in a token and each of these case-sensitive characters are initialized randomly and are updated during model training.

Part-of-Speech Embeddings

Part-of-Speech tags capture the syntactic information of a word in the input sentence. We used Spacy (version 2.0.16) *en_core_web_lg* model[8] that relies on a Convolutional Neural Network trained using OntoNotes to predict part-of-speech tags. The part-of-speech tag representations are initialized with a random embedding and are updated during model training.

Elmo Embeddings

We used the internal representations of BiLM to compute contextual representation of tth token (e_t) in the input sequence as follows:

$$e_t = \gamma * \sum_{j=0}^{L} w_j * h_{t,j}^{LM}$$

$$h_{t,j}^{LM} = \left[\overrightarrow{h_{t,j}^{LM}}, \overleftarrow{h_{t,j}^{LM}} \right] \forall j = 1 \dots L$$

$$h_{t,0}^{LM} = x_t^{LM}$$

where x_t^{LM} indicates the token layer representation of tth word, $h_{t,j}^{LM}$ indicates the context-dependent representations of tth word in the jth layer of BiLM, w indicates the softmax-normalized weight vector and γ is a scaling parameter that allows for task-specific scaling of ELMo representations .

Overall, the token level layer enables the model to generate representations for out-of-vocabulary words using character convolutions and the BiLM layer enables the model to depend on the entire context to generate context-specific representations.

Position Embeddings

Position embeddings capture the relative distance of words in the input sequence with respect to a *target-drug*. Given a sequence with t tokens $S = \{T_1, T_2 \dots T_N\}$ be a sentence with N tokens and $\{T_{d_1}, T_{d_2} \dots T_{d_K}\}$ is a *target-drug* with K tokens. We compute the relative position (P_t) for a token T_t as follows:

$$P_t = \begin{cases} t - d_1, & t < d_1 \\ t - d_K, & t > d_K \\ 0, & d_1 \le t \le d_K \end{cases}$$

Each of these relative positions are initialized with a random embedding and are updated during model training.

Semantic tag Embeddings

The semantic tags are used to differentiate the *target-drug(B-T-Drug), duplicate-target-drug(B-D-Drug), other non-target drugs(B-O-Drug) and other words(O)* in the input sequence. *B-T-Drug* is used to represent tokens in *target-drug* for which we want to extract relations in the current input sequence, *B-D-Drug* is used to represent tokens in *duplicate-target-drug* (a drug in the input sequence with same tokens as *target-drug* but at a different location), *B-O-*

Drug is used to represent tokens of all other drugs in the input sequence and *O* is used to represent all other tokens that does not belong to any of the drugs in the input sequence. The semantic tag representations are initialized with a random embedding and are updated during model training.

REFERENCES

1 Hochreiter S, Urgen Schmidhuber JJ. Long Short-Term Memroy. 1997.

2 Bengio Y, Simard P, Frasconi P. Learning Long-Term Dependencies with Gradient Descent is Difficult. *IEEE Trans Neural Networks* 1994;**5**:157–66.

3 Graves A, Schmidhuber J. Framewise phoneme classification with bidirectional LSTM and other neural network architectures. In: *Neural Networks*. 2005. 602–10.

4 Sutskever I, Martens J, Hinton G. Generating text with recurrent neural networks. In: *Proceedings of the 28th International Conference on Machine Learning, ICML 2011*. 2011. 1017–24.

5 Collobert R, Weston J, Bottou L, *et al.* Natural language processing (almost) from scratch. *J Mach Learn Res* 2011;**12**:2493–537.

6 Lample G, Ballesteros M, Subramanian S, *et al.* Neural architectures for named entity recognition. In: *2016 Conference of the North American Chapter of the Association for Computational Linguistics: Human Language Technologies, NAACL HLT 2016 - Proceedings of the Conference*. 2016. 260–70.

7 Kim Y, Jernite Y, Sontag D, *et al.* Character-Aware neural language models. In: *30th AAAI Conference on Artificial Intelligence, AAAI 2016*. 2016. 2741–9.

8 Honnibal M, Montani I. spaCy2: Natural language understanding with bloom embeddings, convolutional neural networks and incremental parsing. Features. 2017.
