# Supplementary material for: Extraction of Information Related to Drug Safety Surveillance From Electronic Health Record Notes: Joint Modeling of Entities and Relations Using Knowledge-Aware Neural Attentive Models
Source: JMIR Med Inform. 2020 Jul 10;8(7):e18417. doi: 10.2196/18417 (PMC7382020; doi:10.2196/18417)
Supplement: Multimedia Appendix 4 [file medinform_v8i7e18417_app4.pdf]

EXPERIMENTAL SETTINGS

We used 10% of training data as the development dataset for hyperparameter tuning and the remaining 90% for model training. Hyperparameters such as learning rate, dropout was tuned on development dataset whereas the input representation sizes (such as character, parts-of-speech, position embeddings) were empirically decided from our previous experiments on similar datasets.

Table 1. Embedding type sizes

Embedding Type	Dimension Size
Word (w_t)	200
ELMo (e_t)	1024
Character (c_t)	10
Parts-of-Speech (pos_t)	20
Semantic tag (s_t)	30
Position (p_t)	20

Table 1 presents the embeddings types and their sizes used in our experiments.

Table 2. Optimal hyperparameter values of the four methods

Model		Batch Size	Dropout	Learning rate	Hidden Layer Size
joint	Drug Recognition	64	0.3	0.001	100
	Drug-attribute	64	0.2	0.01	100
	Drug-ADE/Reason	64	0.3	0.01	150
ELMo-joint	Drug	64	0.4	0.001	100
	Drug-attribute	64	0.5	0.001	150
	Drug-ADE/Reason	64	0.3	0.01	100
Position-aware-joint	Drug-Attribute	64	0.3	0.01	100
	Drug-ADE/Reason	32	0.3	0.01	150
Knowledge-aware joint	Drug-ADE/Reason	16	0.3	0.001	100

Model regularization is critical for generalization and traditionally, the dropout technique[1] which randomly drops the neuron values during training is used. However, previous research showed standard dropout implementation is ineffective for regularizing recurrent connections, as it disrupts the recurrent neural networks ability to retain long-term dependencies. To address this issue, variational dropout[2], where the same units are dropped across multiple time steps has been proposed. A recent work[3] on biomedical NER studied the impact of variational dropout on NER models and showed variational dropout can significantly benefit in generalizing the models and help in effectively recognizing 'out-of-corpus' entities. Thus, we used variational dropout as regularization method and experimented

with different rates (0.0, 0.1, 0.2, 0.3, 0.4 and 0.5). Furthermore, we experimented with different hidden layer sizes (100, 150, 200) in the Bi-LSTM network.

We chose Adam[4] as our stochastic optimizer with exponential-decayed learning rate. We experimented with several base *learning rates* $(1e^{-2}, 1e^{-3}, 1e^{-4})$ with a decay-rate of 0.96 for every 2000 batches. We used early stopping criteria to terminate model training. Table 2 presents the optimal hyperparameters for each of our models.

REFERENCES

1 Srivastava N, Hinton G, Krizhevsky A, *et al.* Dropout: A simple way to prevent neural networks from overfitting. *J Mach Learn Res* 2014;**15**:1929–58.

2 Kingma DP, Salimans T, Welling M. Variational dropout and the local reparameterization trick. In: *Advances in Neural Information Processing Systems*. 2015. 2575–83.

3 Giorgi JM, Bader GD. Towards reliable named entity recognition in the biomedical domain. *Bioinformatics* 2020;**36**:280–6.

4 Kingma DP, Ba JL. Adam: A method for stochastic optimization. In: *3rd International Conference on Learning Representations, ICLR 2015 - Conference Track Proceedings*. 2015.
